# Metafabric that can cool the human body

**DOI:** 10.1093/nsr/nwab176

**Published:** 2021-09-21

**Authors:** Liping Zhu, Meifang Zhu

**Affiliations:** State Key Laboratory for Modification of Chemical Fibers and Polymer Materials, College of Materials Science and Engineering, Donghua University, China; State Key Laboratory for Modification of Chemical Fibers and Polymer Materials, College of Materials Science and Engineering, Donghua University, China

Along with progress in physics and textile technology, wearable electronics and smart textiles with functions of displaying, supplying power and sensing have been extensively researched [1]. A surge in demand for outdoor protection has also led to the emergence of personal thermal regulation smart textiles, such as radiative cooling fabric [2]. Although mid-infrared transparent radiative cooling fabrics have shown great prospects for indoor cooling and energy saving [3], there remains a challenge in obtaining outdoor daytime radiative cooling fabrics suitable for clothes because of difficulties in getting proper fiber materials and large-scale spinning and weaving [4,5]. In a recent article in *Science*, research teams led by Prof. G. Tao and Prof. Y. Ma in China reported a ‘metafabric’ that can cool the human body by efficiently regulating the spectral properties in the solar radiation band and mid-infrared band at the same time [6]. With a peculiarly designed photonic structure, this metafabric could work even at noon under the strong summer sun. Its flexibility would allow application to the surfaces of all terrestrial objects, such as human bodies, vehicles and buildings.

The reported multi-scale structure of the metafabric, or what the authors called ‘hierarchical-morphology’, is a bilayer structure which has three different levels in terms of spaces and dimensions, providing a full response to UV, VIS-NIR and MIR bands. While the laminated top PTFE layer can strongly reflect UV light from incoming radiation, the TiO_2_ nanoparticle-embedded PLA woven textile not only scatters the entire VIS-NIR light with high efficiency but also has a high MIR absorption (emissivity). Therefore, the fabric can resonantly reflect the solar radiation (0.3–2.5 μm) with reflectance of 92.4% and work as a powerful IR emitter (emissivity ∼94.5%), giving the metafabric excellent radiative cooling capacity compared with commercial fabrics. When wearing this metafabric, the human body can be cooled down to about 3°C lower than when wearing white cotton clothes at noon. Taking advantages of scalable, high-throughput, and fully automatic fabric manufacturing technology, fabric in tens of square meters in area has been produced successfully with great cost-effectiveness, demonstrating the high commercial potential. According to the authors, the fabrication process will introduce only a negligible increment in cost, thus making it potentially suitable for everybody who needs it.

Inspired by interdisciplinary innovations of nanooptics and functional textiles, this research bridges gaps between different disciplines and creates a viable strategy for large-scale manufacture of the metafabric. It overcomes long-standing challenges of applying laboratory-scale radiative cooling fabric to pragmatic scenarios for passive thermal management, with significant enlightenment for innovation and development in the traditional textile industry.

***Conflict of interest statement*.** None declared.
